# Catheter Ablation of Atrial Fibrillation in Patients with Previous Lobectomy or Partial Lung Resection: Long-Term Results of an International Multicenter Study

**DOI:** 10.3390/jcm11061481

**Published:** 2022-03-08

**Authors:** Andrea Demarchi, Giulio Conte, Shih-Ann Chen, Li-Wei Lo, Wei-Tso Chen, Tom De Potter, Peter Geelen, Andrea Sarkozy, Francesco R. Spera, Tobias Reichlin, Laurent Roten, Pascal Defaye, Adrien Carabelli, Serge Boveda, Hamed Bourenane, Lisa Riesinger, Simon Kochhäuser, Gala Caixal, Lluis Mont, Daniel Scherr, Martin Manninger, Francesco Pentimalli, Stefano Cornara, Catherine Klersy, Angelo Auricchio

**Affiliations:** 1 Cardiocentro Ticino Institute, Ente Ospedaliero Cantonale, 6900 Lugano, Switzerland; andrea.demarchi@eoc.ch (A.D.); giulio.conte@eoc.ch (G.C.); 2 School of Medicine, National Yang Ming Chiao Tung University, Taipei Veterans General Hospital, Taipei 11217, Taiwan; epsachen@gmail.com (S.-A.C.); gyrus1975@gmail.com (L.-W.L.); wtchen@vghtpe.gov.tw (W.-T.C.); 3 Cardiovascular Research Institute, National Yang Ming Chiao Tung University, Taipei 11221, Taiwan; 4 Heart Rhythm Center, Division of Cardiology, Department of Medicine, Taipei Veterans General Hospital, Taipei 11217, Taiwan; 5 Cardiovascular Center, Department of Cardiology, Electrophysiology Section, Onze-Lieve-Vrouwziekenhuis (OLV) Hospital, 9300 Aalst, Belgium; tom.de.potter@olvz-aalst.be (T.D.P.); peter.geelen@olvz-aalst.be (P.G.); 6 Cardiology Department, Antwerp University Hospital, 2650 Edegem, Belgium; andreasarkozy@yahoo.ca (A.S.); francesco0802@gmail.com (F.R.S.); 7 University of Antwerp, 2650 Edegem, Belgium; 8 Inselspital, Bern University Hospital, University of Bern, 3012 Bern, Switzerland; tobias.reichlin@insel.ch (T.R.); laurent.roten@insel.ch (L.R.); 9 Cardiology Department, University Hospital of Grenoble Alpes, Grenoble Alpes University, 38043 Grenoble, France; pdefaye@chu-grenoble.fr (P.D.); acarabelli@chu-grenoble.fr (A.C.); 10 Cardiology-Heart Rhythm Management Department, Clinique Pasteur, 31076 Toulouse, France; sboveda@clinique-pasteur.com (S.B.); hamedbourenane@hotmail.com (H.B.); 11 Vrije Universiteit Brussel (VUB), 1050 Brussels, Belgium; 12 Klinik für Kardiologie und Angiologie, 45138 Essen, Germany; lisa.riesinger@uk-essen.de (L.R.); simon.kochhause@uk-essen.de (S.K.); 13 Institut d’Investigacions Biomèdiques August Pi i Sunyer (IDIBAPS), 08036 Barcelona, Spain; gcaixal@clinic.cat (G.C.); lmont@clinic.cat (L.M.); 14 Division of Cardiology, Medical University of Graz, 8036 Graz, Austria; daniel.scherr@medunigraz.at (D.S.); martin.manninger@medunigraz.at (M.M.); 15 S.S. di Elettrofisiologia Cardiaca, S.C. di Cardiologia, Ospedale San Paolo, 17100 Savona, Italy; pentimalli.francesco@gmail.com (F.P.); stefano.cornara@gmail.com (S.C.); 16 Service of Clinical Epidemiology and Biometry, Fondazione IRCCS Policlinico San Matteo, 27100 Pavia, Italy; c.klersy@smatteo.pv.it

**Keywords:** atrial fibrillation, ablation, pulmonary vein stump, pulmonary vein isolation, follow-up, lobectomy, pneumonectomy

## Abstract

Introduction: Data regarding the efficacy of catheter ablation in patients with atrial fibrillation (AF) and patients’ previous history of pulmonary lobectomy/pneumonectomy are scanty. We sought to evaluate the efficacy and long-term follow-up of catheter ablation in this highly selected group of patients. Material and Methods: Twenty consecutive patients (8 females, 40%; median age 65.2 years old) with a history of pneumonectomy/lobectomy and paroxysmal or persistent AF, treated by means of pulmonary vein isolation (PVI) at ten participating centers were included. Procedural success, intra-procedural complications, and AF recurrences were considered. Results: Fifteen patients had a previous lobectomy and five patients had a complete pneumonectomy. A large proportion (65%) of PV stumps were electrically active and represented a source of firing in 20% of cases. PVI was performed by radiofrequency ablation in 13 patients (65%) and by cryoablation in the remaining 7 cases. Over a median follow up of 29.7 months, a total of 7 (33%) AF recurrences were recorded with neither a difference between patients treated with cryoablation or radiofrequency ablation or between the two genders. Conclusions: Catheter ablation by radiofrequency ablation or cryoablation in patients with pulmonary stumps is feasible and safe. Long-term outcomes are favorable, and a similar efficacy of catheter ablation has been noticed in both males and females.

## 1. Introduction

Myocardial sleeves extending between the left atrium and the pulmonary veins are responsible for the spontaneous initiation of paroxysmal atrial fibrillation (AF). Several large studies and registries have shown that electrical isolation of pulmonary veins in patients with paroxysmal and persistent AF is highly effective [1,2]. Pneumonectomy or lobectomy results in a total or partial removal of pulmonary tissue along with the distal segment of the associated pulmonary vein(s), thus leaving one or multiple “pulmonary stump(s)” [3,4]. Two previous studies have shown that pulmonary vein (PV) stumps are electrically active and frequently sites of active firing [5,6], which may be treated by pulmonary vein isolation (PVI). Although acute procedural success rates have been as high as 100%, both studies included a limited number of patients, and only the most recent one included cryoablation for PVI. Long-term efficacy data of PVI in patients with PV stumps are not available. Given the rarity of the clinical scenario of refractory AF in previous pneumonectomy or lobectomy, we sought to collect experience from several international centers, aiming to expand knowledge about procedural efficacy (especially when using cryoablation as an energy source for PVI) and long-term outcomes beyond one year o follow-up.

## 2. Materials and Methods

### 2.1. Study Design

Consecutive adult patients with previous pneumonectomy or lobectomy who underwent PVI due to recurrent drug refractory AF were included in this international multicenter retrospective observational study. All patients younger than 18 years of age, with associated partial anomalous pulmonary venous return, or not consenting to data sharing were excluded. Out of 20 international centers contacted for participation, 11 centers (55%) reported cases treated at their site and provided demographic, interventional, and follow-up data of treated patients. The centers agreeing to participate were: the Cardiocentro Ticino Institute, the Ente Ospedialiero Cantonale, Lugano, Switzerland; the National Yang Ming Chiao Tung University School of Medicine and Taipei Veterans general Hospital, Taipei, Taiwan; the Onze-Lieve-Vrouwziekenhuis (OLV) Hospital, Aalst, Belgium; the Antwerp University Hospital, Edegem, Belgium; the Bern University Hospital, Bern, Switzerland; the Centre Hospitalier Universitaire Grenoble Alpes, Grenoble, France; the Clinique Pasteur, Toulouse, France; the Universiteit Ziekenhuis, Vrije Universiteit Brussel (VUB), Brussels, Belgium; the Klinik für Kardiologie und Angiologie, Essen, Germany; the Hospital Clìnic, Universitat de Barcelona, Barcelona, Spain; the Medical University of Graz, Austria; and the San Paolo Hospital, Savona, Italy. Paroxysmal AF was defined as the occurrence of recurrent episodes of AF self-terminating within seven days; persistent AF was defined as an episode of AF lasting beyond seven days, including episodes terminated by cardioversion after seven days; and long standing persistent AF was defined as continuous AF lasting >twelve months [2]. Physical examinations, medical history, and baseline electrocardiograms (ECG) were obtained for all patients before the invasive procedure. The reasons for the pneumonectomy/lobectomy were collected for each case. This study complies with the Declaration of Helsinki. The study was approved by the Ethics Committee of Canton Ticino in the quality of lead committee (2018-01075/CE 3372), as well as by local institutional review boards. This research did not receive any financial support from funding agencies in the public, commercial, or not-for-profit sectors. 

### 2.2. Catheter Ablation

A CT angiogram of the heart was available before PVI. Anti-arrhythmic drug therapy was discontinued five half-lives before the catheter ablation procedure (except for amiodarone). Intravenous heparin was given with a target activated clotting time of >300 sduring the whole left-sided part of the procedure. The choice between performing a radiofrequency (RF) ablation or a cryoablation was left to the operator. RF ablation was assisted by an electro-anatomical mapping using either a circular mapping catheter (Lasso, Biosense Webster, Irvine, CA, USA; Spiral mapping catheter, Abbott, Chicago, IL, USA) or a multielectrode mapping catheter (PentaRay, Biosense Webster, Irvine, CA, USA/Intellamap Orion, Boston Scientific, Marlborough, MA, USA). RF ablation was performed by using an open irrigated catheter with contact force sensor. Additional lines (roof, superior vena cava, posterior wall electrical isolation) were left to the operator’s discretion. Cryoablation was performed with a single freeze lasting 180 or 240 s; additional freeze was applied until complete isolation was achieved. The choice of the balloon size (23 or 28 mm) and catheter tip length (short vs. long) was left to the operator. Complete vein disconnection was confirmed with pacing maneuvers and/or pharmacological adenosine challenge. The ablation was considered successful in case all PVs, including the stump, were isolated. The PV stump trigger was defined as the recording of earliest electrical activity at or within the PV stump relative to other intracardiac electrograms. Sinus rhythm was restored by electrical cardioversion whenever needed. Major procedural complications were defined as intra- or peri-procedural stroke, systemic embolism, cardiac tamponade, or death.

### 2.3. Clinical Follow-Up

Procedural success was defined as freedom from documented atrial fibrillation, atrial tachycardia, or left atrial flutter lasting ≥30 s after a three-month blanking period (2). Follow-up visits were scheduled according to the practice of each center, with a first follow-up visit after three months and then a yearly visit (unless clinical conditions required more frequent controls). Visits included physical examinations, ECGs, and 24-h Holter recordings. In case the patient missed the scheduled in-person visit, telephone interviews were performed. Additional outpatient clinic visits were immediately performed in case symptoms suggested recurrent arrhythmia. In case symptoms of AF occurred after the blanking period, the patient was offered repeat ablation. Antiarrhythmic drugs were restarted after the catheter ablation for at least three months. Oral anticoagulation was continued after the procedure according to the CHA_2_DS_2_-VASc score. 

### 2.4. Statistical Analysis

Stata 16 (StataCorp, College Station, TX, USA, College Station, TX, USA) was used for all analysis. All tests were two-sided. A *p*-value < 0.05 was considered as statistically significant. Continuous variables are reported as median and 25th–75th percentiles, and categorical variables as counts and percent. Baseline groups were compared by using the Fisher exact test due to the small sample size. Time to event analysis was conducted to assess AF recurrence after the three months blanking period. The reverse Kaplan–Meier method was used to calculate the median follow-up. AF incidence per 100 person-years with its 95% confidence interval (95%CI) was estimated, and the Kaplan–Meier AF recurrence-free survival was then plotted. Due to the fact that the sample size was small no power analysis was performed.

## 3. Results

The demographics and clinical characteristics of the 20 patients enrolled are shown in Table 1. Most of the patients were male (60%) and had previous single lobectomy with concomitant resection of a single PV (75%). Five patients (25%) had a previous unilateral pneumonectomy resulting in partial resection of either the septal or lateral PVs (Table 1). For 18 of the 20 patients (90%), the reason for lung resection was primary cancer. For another patient (5%) it was due to previous infectious diseases, and for the other patient (5%) it was due to previous PV stenosis. Five (25%) patients had multiple ipsilateral stumps.

### 3.1. Procedural Outcome

**Figure 1:**
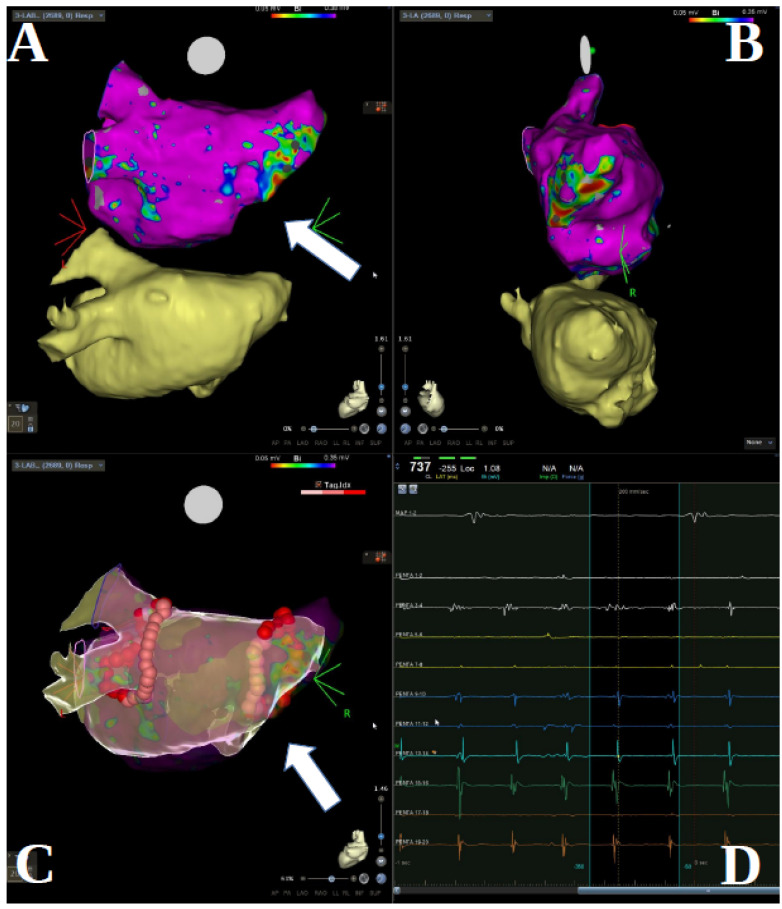
(**A**,**B**) Cardio CT scan and electro-anatomic mapping reconstruction (CARTO) in patient with previous right total pneumectomy, postero-anterior view and latero-lateral view. (**C**) CARTO images of RF PVI in the same patient treated by wide antral isolation. (**D**) Electrogram tracings illustrating atrial arrhythmia inside the right sided pulmonary vein stump as detected by the Pentaray mapping catheter. White arrows point out the stump.

Procedural data are shown in Table 2. The visualization of PV stumps via selective PV angiography or electro-anatomical mapping was always possible (Figure 1, panel A, B). PV stumps were found to be electrically active in 14 patients (70%). In 13 cases (65%) PVI was performed by radiofrequency energy, guided by electro-anatomical mapping, and in seven cases (35%) by cryo-ablation. Isolated antral PVI was performed in eight (61%) patients undergoing RF ablation (Figure 1, panel C); lines at roof, posterior site, and mitral isthmus were added in six cases (46%). Complete PVI was successfully achieved in all patients. No serious procedural adverse events occurred with RF or Cryo.

### 3.2. Arrhythmic Events during Follow-Up

**Figure 2:**
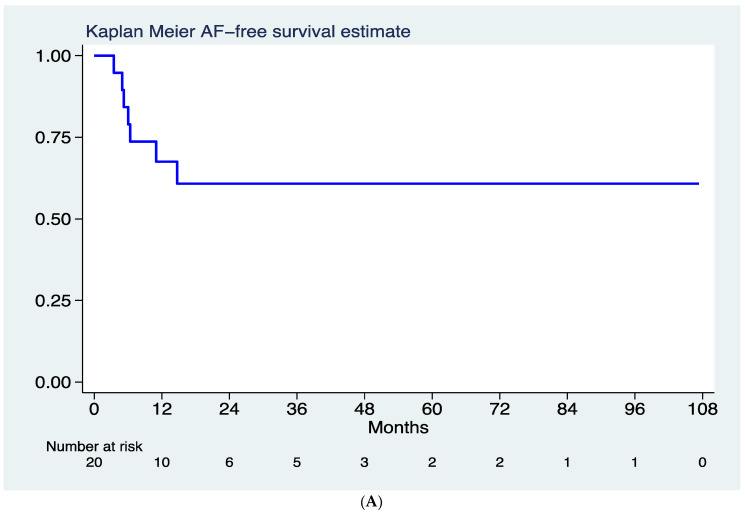

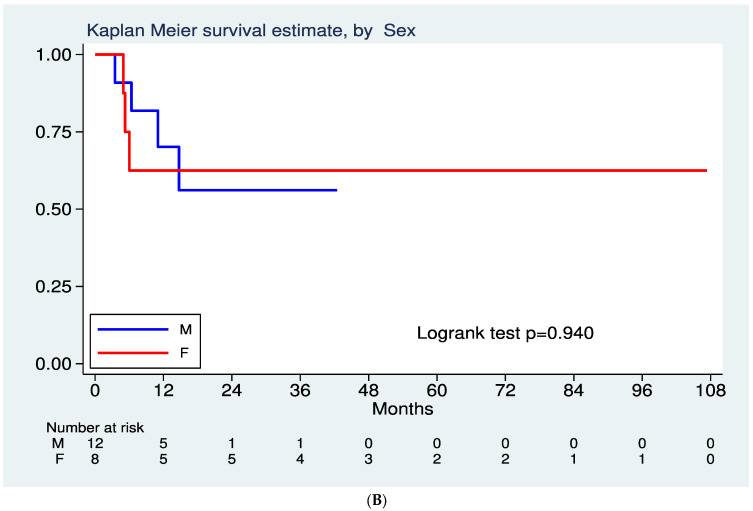
(**A**) Kaplan–Meier curves showing AF recurrence-free survival for follow-up after the blanking period. (**B**) Kaplan–Meier curves showing AF recurrence-free survival for follow-up after the blanking period, after stratification for gender.

Over a median follow up time of 29.7 months (25th–75th 15.2–49.1 months), a total of 7 (35%) AF recurrences were recorded (Figure 2A), corresponding to a rate of 17 recurrences per 100 person-year (95% CI 8–36). One patient was lost on follow-up. Nearly 70% of patients maintained a sinus rhythm at 12 months after a single procedure; at 36 months following follow-up, the AF free rate was 61% (Figure 1). Five patients (25%) underwent a second procedure for documented symptomatic AF after the blanking period. In four of them, ablation was performed using radiofrequency energy. In patients undergoing a redo procedure, the stump reconnection was identified as the reason for arrhythmic recurrence in 60% of cases. Long-term arrhythmic follow-up did not show significant differences regarding arrhythmic recurrences after stratification for gender (Figure 2B). No differences in arrhythmic outcome were noticed after stratification for total pneumectomy vs. lobectomy, nor after stratification for the ablation energy source (RF vs. Cryo). However, a non-significant trend toward higher event incidence was found in patients with single lobectomy (Log rank 0.15). No procedure-related complications were reported during follow-up, nor significant changes in left ventricle ejection fraction. No patients developed indications for pacemaker implantation during long term FU.

## 4. Discussion

The main findings of this international multicenter registry, which enrolled patients at several European and Asian centers, are as follows: (1) pulmonary stumps are often electrically active and may act as trigger sites for AF initiation in a substantial portion of patients; (2) PVI in patients with pulmonary stumps is safe and feasible both with RF energy and cryo-energy; (3) long-term rhythm control of these patients can be successfully achieved with catheter ablation in a good proportion of cases; and (4) no gender-related difference in arrhythmic events was found at follow-up. 

The arrhythmogenicity of pulmonary veins has been demonstrated and resides in their anatomy and embryology [7,8,9]. The autonomic innervation of PVs with abundant ganglions at the epicardial surface of the veno-atrial junction and, above all, the presence of myocardial sleeves contouring PV ostia and extending into PV tissue (up to 25 mm but on average 10 mm) represent the anatomical background for AF onset and maintenance [1,9,10]. Surgical lobectomy and vein ligation/resection leading to denervation of the remnant stump and partial elimination of the myocardial sleeve is expected to eliminate a possible source of AF. However, some case reports [11,12,13] and two single case series [5,6] show that PV stumps are frequently active and a possible source of firing leading to AF initiation [5]. Our results confirmed past observations indicating that in a significant proportion of patients PV stumps are electrically active and a source of atrial firing activity in about one-thirds of the cases (Figure 1, panel D). Superior pulmonary veins are frequently most active in patients with AF, possibly due to a larger extension of myocardial sleeves contouring these veins as compared to inferior veins [10]. In contrast, in our population, a large proportion of inferior stumps (about 50%) were found to be electrically active and 10% showed active firing. 

Pneumonectomy and lobectomy may alter cardiac anatomy. It is known that pneumonectomy may cause a cardiac rotation, diaphragm elevation, and mediastinal shifts [4], resulting in a more complex and possibly riskier transseptal puncture. Ligation and resection of one or more veins may cause the enlargement of the remaining veins, resulting in a more challenging isolation. Also, left sided PV stumps may be hard to differentiate from the left appendage. Finally, blood stagnation inside the stump may favor thrombus formation thus increasing the procedural risk of a thromboembolic event in about 4% of the cases [14]. In contrast to these expected anatomical challenges, 2 case series including 15 [5] and 19 [6] AF patients with previous pneumectomy or lobectomy, respectively, demonstrated the technical feasibility of PVI in all patients. Our study, being the largest case series published so far, not only confirms the technical feasibility of PVI with both RF and cryo-ablation but also expands current knowledge about the use of cryo-ablation in these kinds of patients. In contrast to Kanmanthareddy et al. [5], who reported adverse yet not-well specified procedural events in 13.3% of patients, none of the patients included in our registry reported procedure-related adverse events or complications. Our findings are consistent with Fink et al. [6] who did not observe any procedure-related complications, with an exception made for one case in which a patient suffered a sinus arrest after PVI with subsequent pacemaker implantation.

Patients with pneumectomy and lobectomy affected by drug-refractory AF are significantly under-represented in clinical trials and only recently some case reports and two case series including a dozen of patients have been published [5,6,11,12,13]. Therefore, knowledge about the clinical efficacy of PVI in these highly selected groups of patients is extremely limited. Nearly 70% of our patients treated by means of PVI showed a sinus rhythm after one year. The one-year success rates we observed after the index procedure is similar to the ones observed in AF patients with an intact pulmonary vein anatomy [15,16], but is higher than the one-year success rate of 60% after multiple procedures reported by Fink et al. [6] in 19 patients. As emphasized by these latter authors, in patients with previous pneumonectomy or lobectomy the meticulous anatomical identification of PV stumps, and careful and complete isolation during ablation are key factors for long-term AF suppression. Indeed, Fink et al. [6] showed that in patients with incomplete or unsuccessful stump localization during the index procedure the 1-year arrhythmic event-free rate was as low as 40%. In our multicenter registry, pre-procedural multimodality imaging, including computed tomography angiography or rotational angiography, and conventional and trans-esophageal echocardiography, were always carried out. When PVI was supported by an electro-anatomical mapping system, a CT-merge was always performed. This allowed for precise recognition of vein anatomy and PV stumps. Our study significantly expands current knowledge regarding long-term efficacy of PVI in AF patients with previous lobectomy or pneumonectomy, mainly since the follow-up reported by Fink et al. [6] was limited to 268 days, whereas our median follow-up was at 30 months, and therefore also represents the longest follow-up period published in literature so far. 

It is well known that gender significantly influences clinical presentation, efficacy of the rhythm control strategy, and procedural outcome after catheter ablation. There is a vast literature indicating that women who are referred for AF treatment later, more frequently have atypical symptom presentations, reduced efficacy of the rhythm control strategy including PVI [17,18,19,20], and have higher procedural complication rates [21]. Notably, both Fink et al. and our study showed a higher proportion of female patients (i.e., respectively, 53% and 40% of the entire study population (see Supplementary Materials Table S1)). This finding is in contrast with the numbers found in large European [22] and US [23] catheter ablation registries, in which the female proportion usually does not exceed 30% of the total, as well as with Kanmanthareddy et al. [5] who enrolled only male patients with previous pneumonectomy or lobectomy. A speculative explanation for the larger female proportion of Fink et al. [6] and our study compared to Kanmanthareddy et al. [5] may be the type of lung resection or continental difference. Kanmanthareddy et al. [5] included only US centers, whereas Fink et al. [6] and our study include European and Asian centers. Furthermore, in Kanmanthareddy et al. [5] the vast majority of patients had a total pneumonectomy, which fits the observation by Fink et al. in which patients with total pneumonectomy were more frequently males (67%) versus 35% of males with lobectomy. Notably, no differences in gender-related procedural outcome was noted in our multicenter international patient cohort. In fact, men and women had a comparable rate of arrhythmic recurrences. However, our results need to be interpreted cautiously in consideration of the small sample size. This observation contrasts with large US registry data regarding the outcome for the 2 genders [23] in 21,091 patients, thus representing the largest AF ablation study examining procedural outcomes by gender in the United States. In the US registry [23], women were significantly more likely to be re-hospitalized with AF within one-year after an ablation procedure, but less likely to undergo cardioversion or repeat ablation. The reason for this difference remains unknown and should be investigated.

## 5. Study Limitations

This study has several limitations. It is a small study and thus shares all limitations of observational studies. The patient cohort is admittedly small and has no control group. However, one must bear in mind that drug-refractory AF patients with previous pneumonectomy or lobectomy represent a very small proportion of patients undergoing PVI. This is shown by the fact that 50% of medium-large volume institutions performing AF ablation reported cases fitting the clinical presentation of this study cohort. The lack of systematic monitoring by implantable loop recorders may have led to under-reporting of arrhythmia recurrence. 

## 6. Conclusions

In patients with previous lobectomy or partial resection, pulmonary stumps are electrically active and a possible source of AF initiation. Rhythm control strategy by catheter ablation is feasible and safe with a good long-term outcome. Pulmonary vein isolation by radiofrequency ablation or cryo-ablation shows similar success rates and long-term outcomes. Gender does not have an impact on the outcome. 

## Figures and Tables

**Table 1:** Differences in baseline characteristics between patients treated by means of radiofrequency pulmonary vein isolation or cryoablation.

	All Patients (*n* = 20)	RF Ablation (*n* = 13)	Cryoablation (*n* = 7)	*p* Value
Median Age (years)	65.2 (61.4–72.7)	65.1 (61.1–67.3)	72.7 (63.1–77.2)	*p* = 0.1
CHA_2_DS_2_-VASc	2.0 (1.0–2.5)	2.0 (1.0–2.0)	2.0 (1.0–3.0)	*p =* 0.5
Female sex	8 (40.0%)	5 (38.5%)	3 (42.8%)	*p* = 0.1
LVEF (%)	59% (55–61)	60% (55–62)	60% (56–63)	*p* = 0.2
Clinical History				
Arterial Hypertension	13 (68.4%)	7 (53.8%)	6 (85.7%)	*p* = 0.3
Smoke habit	5 (25.0%)	2 (15.3%)	3 (42.8%)	*p =* 0.2
Diabetes mellitus	1 (5.0%)	1 (7.6%)	0 (0%)	*p =* 0.1
Pre ablation AAD	3 (15.0%)	2 (15.3%)	1 (14.2%)	*p* = 0.1
LAVi (mL/m^2^)	30.7 (26.7–38.0)	32.8 (28–41.0)	27.4 (23.8–31.0)	*p* = 0.4
Type of atrial fibrillation				
Paroxysmal AF	13 (65.0%)	8 (61.5%)	5 (71.4%)	*p* = 0.1
Persistent AF	7 (35.0%)	5 (38.4%)	2 (28.5%)	*p* = 0.1
PV resection				
Pneumectomy	5 (25.0%)	4 (30.7%)	1 (14.2%)	*p* = 0.6
Lobectomy	15 (75.0%)	9 (69.2%)	6 (85.7%)	*p* = 0.5
RSPV	6 (30.0%)	5 (38.4%)	1 (14.2%)	*p* = 0.01
RIPV	4 (20.0%)	0 (0%)	4 (57.1%)	*p* = 0.01
LSPV	4 (20.0%)	3 (23.1%)	1 (14.2%)	*p* = 0.01
LIPV	1 (5.0%)	0 (0%)	1 (14.2%)	*p* = 0.01
Septal PVs	2 (10.0%)	2 (15.3%)	0 (0%)	*p* = 0.6
Lateral PVs	3 (15.0%)	2 (15.3%)	1 (14.2%)	*p* = 0.7

LVEF, left ventricle ejection fraction; AAD, anti-arrhythmic drugs; LAVi, left atrial volume index; AF, atrial fibrillation; RSPV, right superior pulmonary vein; RIPV, right inferior pulmonary vein; LSPV, left superior pulmonary vein; LIPV, left inferior pulmonary vein. *p*-values express the comparison between RF ablation group and the cryoablation group.

**Table 2:** Differences in procedural characteristics between patients treated by means of radiofrequency pulmonary vein isolation or cryoablation.

	All Patients (*n* = 20)	RF Ablation (*n* = 13)	Cryoablation (*n* = 7)	*p* Value
Stump electrical recording	13 (65.0%)	9 (69.2%)	4 (57.1%)	*p* = 0.6
Stump firing	4 (20.0%)	4 (30.7%)	0 (0%)	*p* = 0.2
Balloon tip		-	Long (4 pts) Short (3 pts)	*-*
Balloon size (mm)		-	28 mm (4 pts) 23 mm (3 pts)	-
Irrigated ablation cat.	13 (65%)	13 (100%)	-	-
Procedural time (min)	126 (68.5–173)	134 (101–207)	70 (66–133)	*p =* 0.08
Fluoroscopy time (min)	16.1 (7–27)	17.1 (7.2–39.7)	15.3 (5.6–24.3)	*p* = 0.5
Total ablation time (min)	32.0 (16.3–55.9)	41.2 (18.0–55.9)	17.5 (16.3–18.0)	*p* = 0.3
PVI isolation confirmation technique				
Exit block pacing	3 (15.0%)	3 (23.0%)	0	*p* = 0.5
Entrance block pacing	11 (55.0%)	7 (53.8%)	4 (57.1%)	*p = 0.1*
Adenosine testing	4 (20.0%)	4 (30.7%)	0	*p* = 0.2
EA remapping	4 (20.0%)	4 (30.7%)	-	-

PVI, pulmonary vein isolation; EA, electro-anatomic. *p*-values express the comparison between the RF group and the Cryo group.

## Data Availability

Data available on request due to restrictions eg privacy or ethical. The data presented in this study are available on request from the corresponding author.

## Supplementary Materials

The following supporting information can be downloaded at: https://www.mdpi.com/article/10.3390/jcm11061481/s1, Table S1: Literature cohort comparison.
