# Supplementary material for: Catheter Ablation of Atrial Fibrillation in Patients with Previous Lobectomy or Partial Lung Resection: Long-Term Results of an International Multicenter Study
Source: J Clin Med. 2022 Mar 8;11(6):1481. doi: 10.3390/jcm11061481 (PMC8955984; doi:10.3390/jcm11061481)
Supplement: Supplementary file 1 [file jcm-11-01481-s001.zip › jcm-1578404-supplementary.pdf]

Table S1. Literature cohort comparison.

	**Kanmanthareddy et al.**(J Cardiovasc Electrophysiol 2015;26:385-389)	**Fink et al.** (Pacing Clin Electrophysiol 2020;43:1115-1125)	**This study**
Number of patients	15	19	20
Mean age	63 ± 7	66 ± 10	64 ± 10
Females	0%	53%	40%
**Type of AF**			
Paroxysmal	53%	47%	65%
Persistent	47%	42%	35%
Longstanding persistent	-	11%	-
LVEF	53% ± 5%	>55% (100% of pts)	57% ± 7.1%
**Energy source for PVI**			
RF, manual	100%	68%	65%
RF, robotic	0%	5%	0%
Cryoablation	0%	21%	35%
HIFU	0%	5%	0%
**Procedural data**			
Procedural time	200 ± 33	127.4 ± 48.4	135 ± 76.2
Fluoroscopy time	79 ± 21	17.3 ± 9.2	20.1 ± 17.8
Adverse events	2 (13%)	1 (5%)	0
**Follow-up data**			
Duration of follow-up (days)	No follow-up available	268 (185; 1024)	891 (450 – 1470)
Patients with arrhythmia recurrence	-	63%	35%

AF: atrial fibrillation; LVEF: left ventricle ejection fraction; PVI: pulmonary vein isolation; HIFU: high intensity focused ultrasound
